# Pharmacokinetics/Pharmacodynamics-Based Repositioning of Cefmetazole and Flomoxef in Extended-Spectrum β-Lactamase-Producing Enterobacterales Treatment: An Injectable Carbapenem-Sparing and Outpatient Strategy

**DOI:** 10.3390/antibiotics14080737

**Published:** 2025-07-23

**Authors:** Takahiro Kato, Yusuke Yagi, Takumi Maruyama, Yukihiro Hamada

**Affiliations:** 1 Department of Pharmacy, Kochi Medical School Hospital, Nankoku-City 783-8505, Japan; jm-takkato@kochi-u.ac.jp (T.K.); jm-yyagi@kochi-u.ac.jp (Y.Y.); jm-maruyama.takumi@kochi-u.ac.jp (T.M.); 2 Department of Infection Prevention and Control, Kochi Medical School Hospital, Nankoku-City 783-8505, Japan

**Keywords:** extended-spectrum β-lactamase-producing Enterobacterales, carbapenem-sparing, cefmetazole, flomoxef, pharmacokinetics/pharmacodynamics, outpatient setting

## Abstract

Infections caused by extended-spectrum β-lactamase-producing Enterobacterales (ESBL-Es) pose a significant global threat with notable increases in prevalence worldwide. Carbapenems are often used as the first line of treatment. However, their overuse accelerates resistance development, highlighting the urgent need for clinically viable carbapenem-sparing strategies. Cefmetazole (CMZ) and flomoxef (FMOX) are parenteral antibiotics that are widely used in Japan and have emerged as potential carbapenem alternatives. Repositioning these agents effectively addresses the clinical need for carbapenem-sparing strategies and outpatient ESBL-E management. This review aims to reposition CMZ and FMOX for real-world clinical practice by synthesizing basic research, clinical studies, and pharmacokinetics/pharmacodynamics (PKs/PDs) analyses, which suggest that these agents may be effective in treating ESBL-E infections—particularly urinary tract infections, as evidenced by their minimum inhibitory concentration (MIC) values. The clinical outcomes of these interventions have been comparable to those of carbapenems, which support their role in antimicrobial stewardship. Their PK/PD characteristics emphasize the importance of dose optimization to ensure therapeutic efficacy, whereas recent insights into resistance mechanisms provide a foundation for appropriate use. As novel antibiotic development takes substantial time, revisiting existing options is increasingly important. Notably, the Infectious Diseases Society of America’s 2024 guidance on antimicrobial resistance has omitted CMZ and FMOX, owing to which clinicians have limited guidance on their use, particularly in regions like Japan where these antibiotics are widely employed. By addressing this knowledge gap, the present review offers a comprehensive evaluation of these drugs and highlights their potential as intravenous agents in ESBL-E management. Furthermore, it highlights the ongoing challenge of ensuring effective oral step-down therapy in an outpatient setting to reinforce the global relevance of CMZ and FMOX in a broader treatment framework, underscoring their potential for outpatient administration where clinically appropriate.

## 1. Introduction

Drug-resistant bacterial infections represent a significant global health crisis, contributing to substantial morbidity, mortality, and healthcare expenditures. [1,2]. The worldwide prevalence of antimicrobial resistance among Enterobacterales has increased notably, largely owing to the dissemination of extended-spectrum β-lactamase (ESBL)-producing Enterobacterales (ESBL-Es) [3,4]. Historically, carbapenems have been the cornerstone treatment for ESBL-E infections; however, their widespread use inherently risks the development of further resistance [5]. The recently published Infectious Diseases Society of America’s 2024 guidance on the treatment of antimicrobial-resistant gram-negative infections provides a crucial framework for managing these challenging pathogens [6]; it lacks specific recommendations for cefmetazole (CMZ) and flomoxef (FMOX) [6]. Nevertheless, these agents represent promising carbapenem-sparing alternatives for ESBL-E infections [7]. Effective oral treatment options are advantageous as they facilitate a transition to outpatient therapy, and reduce hospital stays and associated costs. This review aims to assess recent research supporting the use of the injectable antibiotics CMZ and FMOX to treat ESBL-E infections. By collectively presenting basic, clinical, and pharmacokinetics/pharmacodynamics (PKs/PDs) data, we aim to clarify their optimal and rational use and suggest their usage as complementary alternatives to other therapies such as oral carbapenems. Furthermore, this review substantially updates the prior literature by providing a unique focus on the PK/PD-driven repositioning of these agents as crucial carbapenem-sparing and outpatient strategies addressing a critical gap in current guidelines.

## 2. Extended-Spectrum β-Lactamases

**Figure 1:**
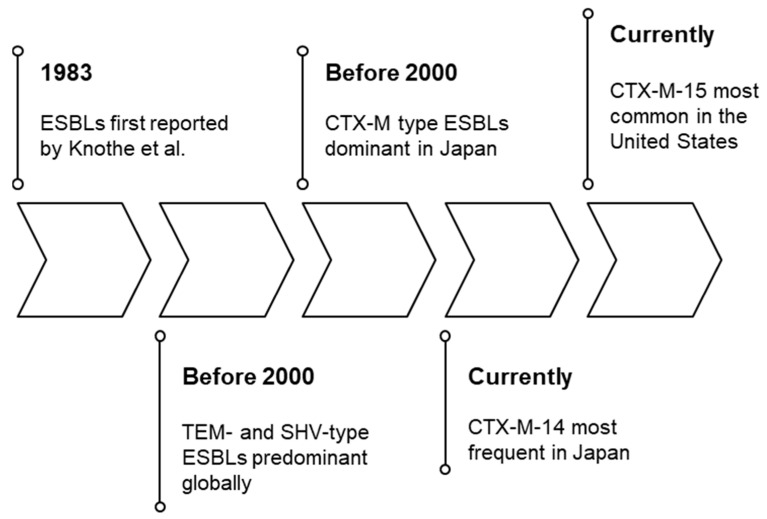
Historical gene circulation of extended-spectrum β-lactamases (ESBLs). The prevalence of ESBLs from their discovery to the present, based on genetic types. ESBLs: extended-spectrum β-lactamases.

ESBLs were first reported by Knothe et al. in 1983. They observed the resistance of *Klebsiella pneumoniae* and *Serratia marcescens* to several cephalosporins [8] and elucidated that this resistance was plasmid mediated. ESBLs are enzymes that inactivate most penicillins, cephalosporins, and aztreonam; however, they are generally susceptible to carbapenems. Although all Gram-negative bacteria potentially harbor ESBL genes, they are most commonly found in *Escherichia coli*, *K. pneumoniae*, *Klebsiella oxytoca*, and *Proteus mirabilis* [9,10,11]. Furthermore, microorganisms housing ESBL genes often harbor additional resistance determinants that extend their resistance to non-β-lactam agents such as fluoroquinolones and trimethoprim-sulfamethoxazole [12,13]. ESBLs include CTX-M enzymes, which exhibit unique hydrolytic capabilities and amino acid substitution variants of TEM and SHV-type β-lactamases [14,15,16,17]. Before 2000, TEM- and SHV-type ESBLs were predominant globally, primarily in *K. pneumoniae* [18]; however, CTX-M type ESBLs were dominant in Japan, primarily in healthcare-associated infections [19,20]. Currently, CTX-M-14 (CTX-M-9 group) is the most frequently detected ESBL in Japan, followed by CTX-M-15 (CTX-M-1 group) [21,22]. In contrast, CTX-M-15 is the most common ESBL in the United States [11] (Figure 1). Understanding the predominant ESBL types in a given region is crucial for guiding empirical and definitive treatment choices.

## 3. Cefmetazole and Flomoxef: Profiles and Properties

The cephamycin CMZ and the oxacephem FMOX are considered important treatment options for ESBL-E infections in Japan because of promising efficacy data which support their use [23,24]. Their profiles are presented in Table 1.

### 3.1. Cefmetazole (CMZ) 

CMZ is a semi-synthetic derivative of cephamycin C within both healthy subjects and patients with impaired renal function, a half-life of approximately 0.8–1.8 h, high urinary excretion rate [25,33], and volume of distribution of 0.165 ± 0.025 L/kg. Its critical characteristic is its high protein-binding rate of 65–79% [29], which significantly impacts plasma concentration measurements and necessitates assays to distinguish the pharmacologically active free (unbound) fraction from the total concentration for accurately guided dosing. Although data on tissue transferability are limited, mean AUC_0–3.5 h_ ratios to plasma are 60% in peritoneal fluid, 36% in peritoneum, and 11% in subcutaneous adipose tissue [46]. Furthermore, decline in renal function prolongs CMZ half-life, necessitating the drug’s removal via dialysis [26]. Halstenson et al. detailed the disposition of cefmetazole in both healthy volunteers and patients with impaired renal function, confirming the need for dose adjustments in the latter group and noting its removal by hemodialysis [26]. CMZ exhibits broad-spectrum activity except against *Pseudomonas aeruginosa* [33]. Generally, CMZ is well-tolerated, and the common adverse effects are gastrointestinal disturbances and potential disulfiram-like reaction; nevertheless, these effects typically reverse upon discontinuation [52,53,54,55]. CMZ can be stored at room temperature for up to 24 h after dissolution (https://image.packageinsert.jp/pdf.php?mode=1&yjcode=6132408E1034 [accessed on 16 July 2025]).

### 3.2. Flomoxef (FMOX) 

FMOX is a broad-spectrum oxacephem antibiotic synthesized in Japan [34]. It exhibits wide activity against Gram-positive, Gram-negative, and anaerobic bacteria, and is stable against ESBLs, which has recently garnered attention [24,35,36,37,38,39]. The elimination half-life for a 1 g intravenous dose was approximately 49.2 min with 85% of the dose excreted unchanged in the urine within 6 h [27]. Ito and Ishigami provided early clinical insights into FMOX’s development and use in Japan [27]. Yamada et al. conducted a phase I clinical study contributing to the understanding of its pharmacokinetic profile [31]. Its volume of distribution for the central and peripheral compartments were approximately 6.6 L and 4.88 L, respectively [28], and it showed a relatively low 35% protein-binding rate [57]. Adverse effects are infrequent but include eosinophilia, elevated transaminase levels, and leukopenia [58,59]. Sato et al. studied FMOX in the pediatric field, providing insights into its use in this population [58]. Chen et al. compared FMOX with latamoxef in treating sepsis and Gram-negative bacteremia, offering further safety data [59]. FMOX exerts a minor and clinically insignificant effect on prothrombin time [32,60]. FMOX can be stored at room temperature for up to 24 h after dissolution (https://www.gifu-upharm.jp/di/mdoc/iform/2g/i3784512010.pdf [accessed on 16 July 2025]).

### 3.3. In Vitro Activity and Pharmacodynamic Considerations for ESBL-E

In Japan, ESBL-producing isolates have shown high susceptibility rates to CMZ and FMOX. Notably, the MIC_90_ values for CMZ, FMOX, and meropenem against ESBL-producing bacteria between 2004 and 2018 were 4 mg/L, 0.5 mg/L, and ≤0.06 mg/L, respectively [21]. Additionally, 57–84% and 97–100% of *E. coli* isolates showed an MIC of < 1 mg/L for CMZ and FMOX, respectively. These proportions were 50–92% and 80–100%, respectively, for *K. pneumoniae* [21]. While in vitro susceptibility may vary subtly by ESBL genotype, comprehensive clinical efficacy data stratified by specific ESBL enzyme types (e.g., CTX-M-15 vs. SHV-12) are generally scarce in the literature, presenting a direction for future research. The notable high resistance of *Enterobacter cloacae* strains to both CMZ and FMOX (MIC of ≥32 mg/L) presents a significant clinical challenge. This highlights the critical need for accurate susceptibility testing prior to treatment initiation, especially in suspected *E. cloacae* infections, to avoid therapeutic failures and promote appropriate carbapenem use [40].

### 3.4. Pharmacokinetics/Pharmacodynamics (Pks/Pds) of Cefmetazole

The primary PK/PD index associated with β-lactams efficacy is fT>MIC, which is the percentage of the dosing interval during which the free drug concentration remains above the MIC. For high-burden infections like complicated UTIs, the inoculum effect can influence the effective MIC, potentially requiring a higher fT>MIC target for optimal clinical outcomes. As CMZ shows a high protein-binding rate, the parameters based on free concentration are important when considering its effects on clearance and distribution volume [30]. Namiki et al. conducted a PK/PD analysis using unbound cefmetazole concentrations, emphasizing the significance of the free drug fraction for accurate dosing in patients with ESBL-E infections [30]. Hence, a target fT>MIC of > 70% in vivo has been proposed for CMZ for treating complicated urinary tract infections (cUTIs) caused by ESBL-E [7]. Actual clinical measurement of free drug concentration is difficult, so clinical differences need further consideration. Other PK/PD indices such as the ratio of the maximum free concentration to the MIC (fCmax/MIC) or the area under the free drug concentration–time curve to MIC ratio (fAUC/MIC) may be relevant for different infection types, although these targets are less established for CMZ [42]. Hamada et al. conducted a retrospective study, in which a 1 h intravenous infusion of 1 g of cefmetazole every 8 h effectively treated invasive urinary tract infections caused by ESBL-producing *E. coli* with an MIC of ≤4 mg/L in patients with normal renal function [43]. Additionally, Hamada et al. performed a Monte Carlo simulation and determined that adjusted dosing regimens achieved a 90% probability of target attainment at a PK/PD breakpoint of 4 mg/L in patients with varying renal function [45]. Similarly, Namiki et al. showed that a 1 g dose administered post-dialysis to hemodialysis patients was effective against strains with an MIC of ≤ 4 mg/L [30]. In contrast, an in vitro chemostat model has suggested that a standard regimen of 1 g every 6 h may be insufficient for strains with MICs of 4–16 mg/L [21]. For strains with an MIC of 8 mg/L, a higher dose of 2 g every 6 h should be considered. This underscores the importance of MIC-guided therapy and potential dose escalation, especially for more resistant strains, to optimize clinical outcomes and ensure adequate drug exposure.

### 3.5. Pharmacokinetics/Pharmacodynamics (Pks/Pds) of Flomoxef

A dosing regimen of 1 g every 8 h (2 h infusion) or 1 g every 6 h (1 h infusion) of FMOX achieves an fT>MIC of >70% [42]. This fT>MIC target is based on the free drug concentration, reflecting the unbound and pharmacologically active portion of FMOX. An in vitro model confirmed that the dosage of 1 g every 6 h provided sufficient bactericidal effect against strains with MICs of ≤4 mg/L [21]. FMOX shows bactericidal potential against susceptible ESBL-E supported by in vitro findings and fT>MIC target. Notably, FMOX exhibited good tissue penetration. A study in patients undergoing gastrointestinal surgery showed that a dosing regimen of 1 g every 6–8 h achieved an fT>MIC of >40% in ascitic fluid, peritoneal tissue, and subcutaneous fat, provided the MIC was 1 mg/L [49]. This study by Hirano et al. provided valuable insights into FMOX distribution in surgical patients, suggesting its potential for treating intra-abdominal infections [44]. In patients with benign prostatic hyperplasia, a regimen of 1 g every 6 h achieved an fT>MIC of >90% in prostatic tissue at an MIC of 0.5 mg/L [44]. Nakamura et al. highlighted the favorable tissue penetration into prostatic tissue, supporting its use for prostatic infections [28]. These findings support the potential usage of FMOX to treat various ESBL-E infections in addition to those of the urinary tract.

## 4. Clinical Evidence and Comparison with Other Therapeutic Options

### 4.1. Cefmetazole and Flomoxef vs. Carbapenems

Several studies have compared the effects CMZ and FMOX with those of carbapenems. For example, a prospective study on ESBL-producing *E. coli*-induced urinary tract infections found no significant difference in clinical effectiveness or mortality between CMZ and meropenem [47]. This study, involving 117 patients, specifically highlighted that CMZ maintained comparable efficacy to meropenem for these infections. Microbiological eradication report varied but observed clinical effectiveness and PK/PD targets generally indicate bactericidal activity against susceptible isolates. Similarly, retrospective studies on bacteremia have shown no difference in 90-day mortality rates between CMZ (17.5%) and carbapenems (16.7%) [48]. Another study reported elucidated that for patients with ESBL-E bacteremia, the all-cause 30-day mortality rates were 13.5% in the CMZ/FMOX group and 10.9% in the carbapenem group in propensity-score-matched cohorts, showing no statistically significant difference [41]. A retrospective study found higher mortality with FMOX in bacteremia cases involving isolates with MICs of 2–8 mg/L, suggesting standard dosing may be inadequate. This may reflect the severity of bacteremia or undetected resistance or virulence factors. Despite limitations like confounding, the findings underscore the importance of MIC-guided therapy and cautious FMOX use when carbapenems are an option. [50,56,60]. This highlights the importance of MIC-guided therapy. These findings suggest that CMZ and FMOX are effective carbapenem-sparing options, although caution is warranted in cases of severe infections or when treating isolates with relatively high MICs.

### 4.2. Other Therapeutic Options 

**Figure 2:**
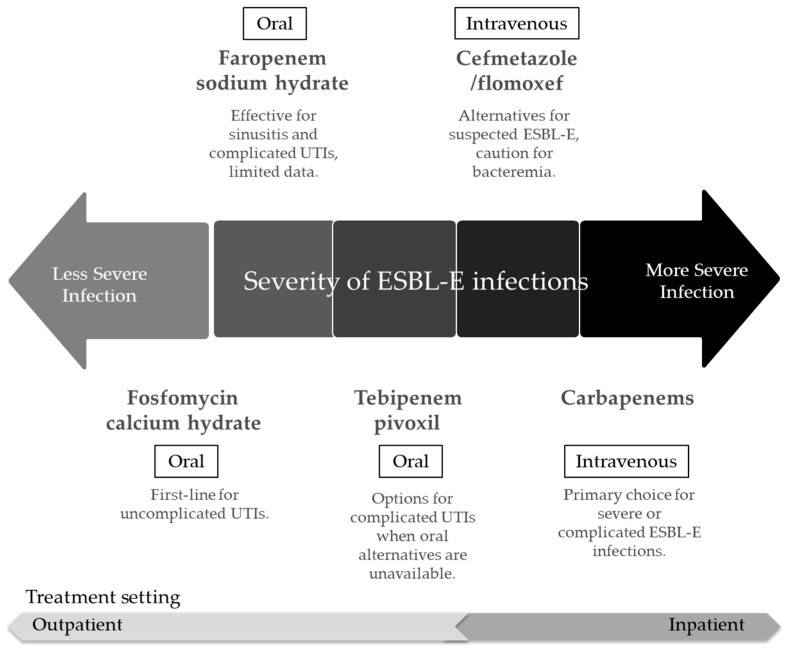
Treatment options and strategies for extended-spectrum β-lactamase-producing Enterobacterales (ESBL-Es) infections. Antibiotic treatment selection based on severity and treatment setting for ESBL-E infections. ESBL-E: extended-spectrum β-lactamase-producing Enterobacterales; UTIs: urinary tract infections.

In addition to CMZ and FMOX, several other viable agents have been reported. For example, tebipenem pivoxil and faropenem sodium hydrate are oral carbapenem/penem antibiotics that are stable against ESBLs and are valuable for outpatient or switch therapy [61]. Tebipenem pivoxil, approved in Japan and recently pending approval in the United States for complicated urinary tract infections, is an oral prodrug that has shown non-inferiority to intravenous ertapenem for cUTIs and pyelonephritis caused by resistant pathogens such as ESBL-E [62]. Furthermore, Monte Carlo simulation has identified the optimal renal-adjusted oral tebipenem dosing regimens for Japanese adults with ESBL-E urinary tract infections, which offer a potentially effective outpatient treatment option [63]. Additionally, faropenem sodium hydrate has been suggested for ESBL-E infections in some regions, including Japan, but the currently available clinical data are limited [64], and reports of emerging resistance has raised concerns with respect to potential cross-resistance to carbapenems [65,66]. Fosfomycin calcium hydrate has been recommended as a first-line agent for treating uncomplicated UTIs [67]. Although carbapenems remain the primary choice for severe infections, alternatives such as β-lactam/β-lactamase inhibitor combinations may be considered [68,69,70] (Figure 2).

## 5. Challenge of Resistance and Future Perspectives

Resistance to CMZ in *E. coli* appears to be mediated by a reversible porin-dependent mechanism. CMZ exposure leads to decreased expression of the porin channels OmpF and OmpC, which results in resistance. Notably, this resistance was sometimes reversed upon drug removal and was suppressed by the β-lactamase inhibitor relebactam, which restored OmpF expression [51,71]. Future research on CMZ resistance should focus on definitively characterizing the prevalence and clinical impact of this porin-mediated resistance in vivo and exploring the potential for co-administration with β-lactamase inhibitors to overcome such mechanisms. The mechanisms underlying FMOX resistance remain uninvestigated. Closing this knowledge gap on resistance mechanisms is crucial for preserving FMOX efficacy and guiding clinical decisions amid evolving resistance. Future studies are needed to identify the genetic and phenotypic determinants of FMOX resistance, including potential enzymatic hydrolysis or efflux pump systems. Furthermore, detailed clinical data on the rate of on-therapy resistance development to CMZ or FMOX during treatment of ESBL-E infections, especially in direct comparison to carbapenems, are not readily available in the current literature and represent an important area for future investigation. Hence, future studies must combine the investigation of novel β-lactamase inhibitors with robust antimicrobial stewardship and a deeper understanding of resistance mechanisms. Consequently, advanced genomic and metagenomic sequencing will be crucial for real-time surveillance and elucidating the diversity and transmission of resistance genes [72,73]. 

## 6. Conclusions

Antimicrobial options for ESBL-E infections remain limited. However, the strategic rediscovery and optimized use of older antibiotics like CMZ and FMOX represent a valuable approach to combat the antimicrobial resistance crisis. These agents serve as important carbapenem-sparing alternatives—particularly for less severe infections such as urinary tract infections, and should always be used with susceptibility testing guidance, ideally against isolates with low MICs (e.g., ≤4 mg/L). Their parenteral nature allows for initial stabilization in the inpatient setting, followed by continuation in outpatient intravenous administration programs facilitating early discharge and reduced healthcare burden. Outpatient intravenous CMZ/FMOX needs strong infrastructure like infusion centers or home health and development of long-acting formulations with long-term stability. Challenges include patient eligibility, insurance, nursing access, and patient education/monitoring. Furthermore, viable treatment alternatives for ESBL-E infections include oral tebipenem pivoxil for outpatient therapy, fosfomycin calcium hydrate for simple cystitis, and β-lactam/β-lactamase inhibitors for complex cases. Local ESBL epidemiology and resistance patterns are crucial when applying the pharmacological principles and in vitro data of CMZ and FMOX. Although the efficacy of CMZ and FMOX requires further validation for all clinical syndromes, their appropriate use can help reduce carbapenem consumption and mitigate the selection pressure that leads to further resistance.

## Figures and Tables

**Table 1:** Summary of pharmacokinetics and pharmacodynamics characteristics of cefmetazole and flomoxef.

Category	Characteristic	Cefmetazole (CMZ)	Flomoxef (FMOX)
Pharmacokinetics (PKs)	Half-life	Approx. 0.8–1.8 h [25,26]. Prolonged as renal function declines [26].	Approx. 49.2 min following a 1 g intravenous dose [27].
	Volume of distribution	0.165 ± 0.025 L/kg [25].	Central compartment: approximately 6.6 L [28]. Peripheral compartment: approximately 4.88 L [28].
	Protein binding	High: 65–79% [29]. Free (unbound) fraction is pharmacologically active [29,30].	Low: 35% [31].
	Excretion	High urinary excretion rate (approx. 87% in 24 h) [25].	85% of the dose is excreted unchanged in urine within 6 h [27].
	Effect of renal impairment/dialysis	Half-life is prolonged in patients with impaired renal function [26]. Readily removed by hemodialysis [26,30].	Dosing adjustments may be necessary in renal insufficiency [32].
Pharmacodynamics (PDs)	Spectrum of activity	Broad-spectrum cephamycin [33]. Not active against *Pseudomonas aeruginosa* [33].	Broad-spectrum oxacephem against Gram-positive, Gram-negative, and anaerobic bacteria [34,35]. Stable against ESBL-E [24,35,36,37,38,39].
	In vitro activity (vs. ESBL-E)	Minimum inhibitory concentration (MIC_90_): 4 mg/L [21]. Susceptibility: For *Escherichia coli*, 57–84%; MIC of 1 mg/L [21]. For *Klebsiella pneumoniae*, 50–92%; MIC of 1 mg/L [21]. *Enterobacter cloacae* often exhibits high resistance (MIC ≥32 mg/L) [40].	MIC_90_: 0.5 mg/L [21]. Susceptibility: For *E. coli*, 97–100%; MIC of 1 mg/L [21]. For *K. pneumoniae*, 80–100%; MIC of 1 mg/L [21]. *E. cloacae* often exhibits high resistance (MIC ≥32 mg/L) [40].
PK/PD	Primary PK/PD index	Percentage of dosing interval during which the free drug concentration remains above the MIC (fTMIC) [30]. Clinical application is a challenge.	Percentage of the dosing interval during which the drug concentration remains above TMIC [41,42].
	PK/PD target	TMIC > 70% has been proposed for complicated urinary tract infections (cUTIs) [43].	TMIC > 70% [41,44].
	Dosing and target attainment	Adjusted dosing achieves 90% PTA for an MIC of 4 mg/L [45]. Dose of 2 g q6h is needed for strains with MIC of 8 mg/L [45]. In hemodialysis patients, a 1 g post-dialysis dose is effective against strains with MIC ≤4 mg/L [30].	1 g q8h (2 h infusion) or 1 g q6h (1 h infusion) achieves TMIC > 70% [41,44]. 1 g q6h provides sufficient bactericidal effect against strains with MICs ≤4 mg/L [21].
	Tissue penetration	Free concentration is important for distribution volume [30]. Mean AUC_0–3.5 h_ ratios to plasma are 60% in peritoneal fluid, 36% in peritoneum, and 11% in subcutaneous adipose tissue. [46].	Good tissue penetration. Achieves PK/PD targets in ascitic fluid, peritoneal tissue, subcutaneous fat, and prostatic tissue [28,42].
Clinical application and precautions	Clinical efficacy (vs. carbapenems for ESBL-E)	No significant difference in clinical effectiveness or mortality for *E. coli*-related UTIs [47]. No significant difference in 90-day or 30-day mortality for bacteremia [36,48].	A propensity-score-matched study showed no difference in 30-day mortality for bacteremia [36]. One retrospective study reported a higher mortality rate with FMOX vs. carbapenems, particularly for isolates with MICs of 2–8 mg/L [49,50].
	Precautions and considerations	Considered a carbapenem-sparing alternative [23]. Caution is warranted in severe infections or for isolates with high MICs [47].	Use should be guided by MIC testing, especially in severe infections like bacteremia [49,50]. A valuable carbapenem-sparing option [36].
	Resistance mechanisms	Resistance in *E. coli* is linked to a reversible porin-dependent mechanism (decreased OmpF and OmpC expression) [51].	Mechanisms underlying resistance to FMOX have not been investigated.
Safety	Adverse effects	Generally well-tolerated. Common effects include gastrointestinal disturbances [52,53,54]. These are typically reversible [52,53,54,55].	Adverse effects are infrequent but can include eosinophilia, elevated transaminases, and leukopenia [56,57,58].
	Specific warnings	Potential for a disulfiram-like reaction with alcohol [52,53,54,55].	Exhibits minor and clinically insignificant effect on prothrombin time [32,59].

ESBL-Es: extended-spectrum β-lactamase-producing Enterobacterales; MIC: minimum inhibitory concentration; TMIC: time above minimum inhibitory concentration; UTIs: urinary tract infections; PTA: probability of target attainment; AUC: area under the drug concentration–time curve.

## Data Availability

No new data were created or analyzed in this study. Data sharing is not applicable to this article.

## Abbreviations

The following abbreviations are used in this manuscript:

ESBL-Es	extended-spectrum β-lactamase-producing Enterobacterales
MIC	minimum inhibitory concentration
TMIC	time above minimum inhibitory concentration
UTIs	urinary tract infections
PTA	probability of target attainment
CMZ	cefmetazole
FMOX	flomoxef
PKs/PDs	pharmacokinetics/pharmacodynamics
AUC	area under the drug concentration–time curve
